# Taking a Bad Turn: Compromised DNA Damage Response in Leukemia

**DOI:** 10.3390/cells6020011

**Published:** 2017-05-04

**Authors:** Nadine Nilles, Birthe Fahrenkrog

**Affiliations:** Institute for Molecular Biology and Medicine, Université Libre de Bruxelles, 6041 Charleroi, Belgium; nnilles@ulb.ac.be

**Keywords:** DNA damage response, leukemia, double-strand break repair, non-homologous end joining, homologous recombination

## Abstract

Genomic integrity is of outmost importance for the survival at the cellular and the organismal level and key to human health. To ensure the integrity of their DNA, cells have evolved maintenance programs collectively known as the DNA damage response. Particularly challenging for genome integrity are DNA double-strand breaks (DSB) and defects in their repair are often associated with human disease, including leukemia. Defective DSB repair may not only be disease-causing, but further contribute to poor treatment outcome and poor prognosis in leukemia. Here, we review current insight into altered DSB repair mechanisms identified in leukemia. While DSB repair is somewhat compromised in all leukemic subtypes, certain key players of DSB repair are particularly targeted: DNA-dependent protein kinase (DNA-PK) and Ku70/80 in the non-homologous end-joining pathway, as well as Rad51 and breast cancer 1/2 (BRCA1/2), key players in homologous recombination. Defects in leukemia-related DSB repair may not only arise from dysfunctional repair components, but also indirectly from mutations in key regulators of gene expression and/or chromatin structure, such as p53, the Kirsten ras oncogene (K-RAS), and isocitrate dehydrogenase 1 and 2 (IDH1/2). A detailed understanding of the basis for defective DNA damage response (DDR) mechanisms for each leukemia subtype may allow to further develop new treatment methods to improve treatment outcome and prognosis for patients.

## 1. Introduction

Genome integrity is of outmost importance for cell survival and for the wellbeing of any organism. Every organism is constantly exposed to genotoxic stress and every single cell is consequently subjected to tens of thousands of DNA lesions every day, which thus need to be repaired to preserve genomic integrity [1]. To repair DNA lesions, cells have evolved specialized, sophisticated repair pathways, which are globally termed DNA damage response (DDR) [2,3,4]. DDR is a highly orchestrated signaling cascade closely linked to the cell cycle, i.e., the activation of cell cycle checkpoints and arrest and the resumption of cell cycle progression when DNA damage has been removed in full [5,6]. Persistence of damage will direct cells towards cellular senescence or apoptosis. DDR signaling is specific to the type of damage that occurs and can be roughly discriminated between excision repair (ER), single-strand break (SSB) repair and double-strand break (DSB) repair. In particular, DSBs, which may arise from errors in DNA metabolism or genotoxic insults, such as chemotherapeutic drugs, challenge the integrity of our genomes and may lead to mutagenesis. DSBs are processed by either non-homologous end joining (NHEJ) or homology directed repair (HDR), namely homologous recombination (HR) and single strand annealing (SSA). Homology-independent repair occurs mostly during G1, while homology-dependent repair utilizes sister chromatids as template for repair for which reason it exclusively takes place during late S and G2 phases of the cell cycle [2,3,5].

The central role of the DDR in human physiology is indicated by a broad spectrum of disorders described in individuals carrying mutations in DDR genes, such as ataxia telangiectasia mutated (ATM) and Nijmegen breakage syndrome (NBS) [7,8]. These DDR genetic syndromes primarily affect tissue homeostasis, for example of the nervous, immune, and reproductive system, and they can lead to premature aging or may predispose affected individuals to cancer development [9]. The importance of DNA damage in the context of cancer is nicely reflected by a quotation from Michael Kastan: “DNA damage causes cancer, is used to treat cancer, and contributes to the side-effects of treating cancer” [10]. Tumor cells are typically characterized by the loss of one or more DDR pathways, which needs to be compensated by the remaining, intact DDR pathways. Precancerous cells, on the contrary, oppose an increased number of DNA lesions by boosted DDR activity [5]. Cancer therapy through radio- and chemotherapy aims at bringing about an irreparable number of DNA breaks to activate DDR in the tumor cells and direct them into apoptosis. Due to the higher proliferation rate of the tumor cells, they are more sensitive to this treatment as compared to normal cells, and despite the lack of specificity and a plethora of side effects, radio- and chemotherapy are often efficient [11]. Numerous tumors, however, are resistant to therapy, in particular chemotherapy, often because of mutations in DDR genes. Cancers with mutations in DDR genes are critically associated with poor treatment and survival prognosis, both in solid tumors and in leukemia [10,11,12]. Here we review current knowledge about aberrant DDR signaling in the context of cancer, with a particular emphasis on aberrant DDR in leukemia.

## 2. DNA Double-Strand Break Repair

**Figure 1:**
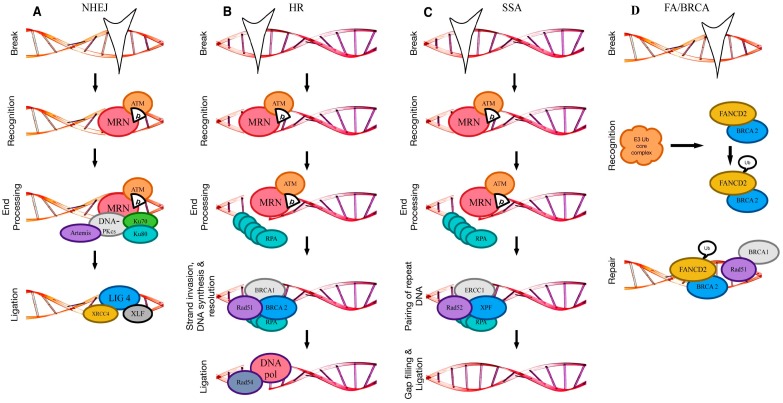
Schematic presentation of the major steps of the different double-strand break (DSB) repair pathways. (**A**) Non-homologous end joining starts with break recognition by the Mre11-Rad50-Nbs1 (MRN) complex and subsequent phosphorylation of and by ataxia telangiectasia mutated (ATM) to signal the break and recruit further repair components. End processing is mediated by the DNA-dependent protein kinase catalytic subunit (DNA-PKcs)/Ku70/80 complex and Artemis is recruited to prepare the DNA end for ligation, which is performed by the XRCC4-like factor (XLF)/ X-ray repair cross-complementing protein 4 (XRCC4)/DNA ligase 4 (LIG4) complex. (**B**) Homologous recombination (HR) equally starts with break recognition by the MRN complex and subsequent phosphorylation of and by ATM. Mre11 and other nucleases form single-strand DNA (ssDNA) overhangs that become coated by replication protein A (RPA). Strand invasion, DNA synthesis and resolution is mediated by Rad51, breast cancer 1/2 (BRCA1/2) and ligation by the DNA polymerase and Rad54. (**C**) Single-strand annealing starts alike HR, but after end processing Rad52 simply anneals the ssDNA ends and the non-homologous tails are cut off by excision repair 1 (ERCC1) and Xeroderma pigmentosum complementation group F protein (XPF). (**D**) The Fanconi anemia pathway starts with break recognition by the E3 ubiquitin core complex that ubiquitylates Fanconi anemia complementation group protein D2 (FANCD2). The FANCD2/BRCA2 complex can relocalize to the break and act together with components of the HR machinery to repair the break. SSA: single-strand annealing; NHEJ: non-homologous end joining.

DNA double-strand breaks (DSBs) may arise when DNA replication is corroborated by, for example, the presence of SSBs, DNA nicks or DNA cross-links. Exposition to ionizing radiation or chemotherapeutic agents may trigger DSBs extrinsically [13]. Two major pathways are critical for the repair of DSBs: NHEJ and HR. Classical NHEJ (c-NHEJ), the major NHEJ variant, is an error-prone repair pathway and occurs throughout the entire cell cycle, mainly during G1, as no homologous sequence is required as a template for repair. HR, by contrast, is much more faithful and can only be accomplished during late S/G2 after DNA replication, as it relies on the sister chromatid for an accurate repair of a DSB [14]. Another homology-based repair pathway is SSA, which, as with HR, can only take place after DNA replication [15]. A minor and ill-defined NHEJ variant, known as alternative NHEJ (alt-NHEJ) or microhomology (MH)-mediated end joining (MMEJ) relies on MH annealing and appears mechanistically closer to HR than NHEJ [16]. Indeed, the presence of defective HR factors increases the frequency of MMEJ, in yeast and humans [17,18]. Pathway choice is hence largely influenced by the cell cycle state, but further by, for example, the position of the break within the nucleus, the chromatin state, i.e., heterochromatic versus euchromatic regions, and the epigenetic state, (in particular DNA methylation), as well as by the condensation state of the chromosomes [6,19,20,21]. Pathway choice is largely controlled by the Mre11-Rad50-Nbs1 (MRN) complex (Figure 1A,B), which acts as a DSB sensor, as well as co-activators of the three DDR-related cell cycle checkpoints (i.e., the G1/S checkpoint, which prevents cells from entering S phase, the intra-S checkpoint inhibiting replication during S phase, and the G2/M checkpoint, which prevents damaged cells from entering mitosis), and DSB effector proteins in NHEJ and HR [22]. Sensing of a DSB by the MRN complex leads to the recruitment and activation of the signaling kinases ataxia telangiectasia mutated protein (ATM; Figure 1A,B), the ATM and Rad3-related kinase (ATR), and the DNA-dependent protein kinase (DNA-PK), which phosphorylate a specific serine residue of the histone variant H2A.X at the break site and its flanking regions [23,24,25]. Phosphorylation of H2A.X leads in turn to further recruitment and phosphorylation of MRN and other pathway-specific DNA damage mediators, such as p53-binding protein 1 (53BP1; NHEJ) and breast cancer 1 (BRCA1; HR) [22,26].

### 2.1. Non-Homologous End Joining

Repair of a DSB by NHEJ is initiated by the rapid binding of the Ku heterodimer, formed by the two ATP-dependent DNA helicases Ku70 and Ku80 (Figure 1A), to the ends of DNA [27,28,29]. Ku recruitment is followed by the recruitment and activation of the DNA-dependent protein kinase catalytic subunit (DNA-PKcs), an ATM-related kinase. DNA-PKcs keep the broken DNA ends in close proximity and recruit end-processing factors, such as Artemis (Figure 1A), which prepares the DNA ends for religation by the X-ray repair cross-complementing protein 4 (XRCC4)–XRCC4-like factor (XLF)–DNA ligase 4 (LIG4) complex [22,26,30,31]. Artemis’ endonuclease activity is activated by autophosphorylation of the DNA-PKcs and a subsequent conformational change [30]. Religation is finalized by the XRCC4/XLF/LIG4 complex. XRCC4 stabilizes LIG4 and XLF (also known as non-homologous end joining factor 1 (NHEJ1)), stimulating its ligation ability [14,27].

### 2.2. Homologous Recombination

As outlined above, the defining feature of HR is the utilization of the sister chromatid to guide repair of a DSB during the S and G2 phases of the cell cycle [32]. During HR, DSBs are resected to give extensive 3′ single-strand DNA (ssDNA) overhangs on each side of the break [33,34]. These 3′ ssDNA overhangs are generated by exonucleolytic processing mediated by several nucleases or nuclease complexes, including Mre11 [35,36], Bloom’s syndrome helicase (BLM), MRN/CtBP-interacting protein (CtIP), exonuclease 1 (EXO1) [37] and DNA replication ATP-dependent helicase (DNA2) [37]. The 3′ ssDNA overhangs serve as a substrate for the HR-specific ssDNA-binding factor replication protein A (RPA) and Rad51 (Figure 1B) (reviewed in [25,38]), which ensures that the DSB will be repaired via HR [39,40]. It also reduces the efficiency of NHEJ, because the Ku70/80 heterodimer has poor affinity for ssDNA [39]. RPA coats and stabilizes the ssDNA overhangs [25,41] and will be replaced by the key facilitator of homologous recombination, Rad51, which displaces RPA to form a so-called Rad51–ssDNA nucleofilament. Rad51 nucleofilament assembly requires the help of mediator proteins, such as breast cancer 1 and 2 (BRCA1 and BRCA2) and several Rad51 paralogues [41,42], but the exact mechanisms that underlie this process are only partially understood. The Rad51-ssDNA nucleofilament searches for DNA sequences similar to the 3′ overhang and will invade the recipient DNA strand, i.e., the sister chromatid. During strand invasion, a so-called displacement loop (D-loop) is formed [41]. Rad54 next catalyzes the removal of Rad51 from the ssDNA to give access to the DNA polymerase, which initiates synthesis of new DNA to replace the DNA surrounding the former break site [43]. Annealing to the new DNA strand leads to the formation of two crossed strands, the so-called Holliday junctions (HJs). HJs are resolved by resection and, after gap-filling DNA synthesis and ligation, give rise to repaired strands. Depending on how the junctions are resected, this leads of either crossover or non-crossover products [41]. HR plays an important role not only in DSB repair, but also in meiosis (reviewed in [41]). 

Various HR factors are implicated in cancer. For example, mutations in the *RAD54* homolog *RAD54B* and in CtIP are associated with lymphomas and colon cancer [44,45], whereas BRCA1 is frequently associated with breast cancer [46]. In fact, BRCA1 expression is often decreased in sporadic basal-like breast cancer, which represents around 90% of the total breast cancer cases [46]. Similar to BRCA1, BRCA2 dysfunction is commonly found in breast and ovarian cancer [47,48]. Rad51 is frequently over-expressed in soft tissue sarcoma (STS) with resistance to doxorubicin treatment [49], and Rad51B was found implicated in lipoma and uterine leiomyoma [50]. RECQL4, a DNA helicase of the ATP-dependent helicases RecQ family, has been associated with basal and squamous cells skin carcinomas as well as with osteosarcoma [51]. Also, BLM, the Werner syndrome RecQ like helicase (WRN), and NBS1 are implicated in different types of cancer [7,51,52].

### 2.3. Single-Strand Annealing

SSA, similar to HR, is restricted to the late S and G2 phases. It does not require the presence of the sister chromatid, but it is initiated by end resection [52], which can only occur in S/G2, as it in part depends on cyclin-dependent activation of CtIP [53,54]. Rad52 is responsible for the annealing of the flanking repeats that result from the end resection [55,56]. Excision repair 1 (ERCC1) in a complex together with Xeroderma pigmentosum complementation group F protein (XPF) then removes the non-homologous 3′ ssDNA tails [57]. Polymerases and ligases are responsible for the final steps, gap filling and ligation, but the exact players remain still poorly established [52].

### 2.4. Alternative Non-Homologous End Joining/Microhomology-Mediated End Joining (Alt-NHEJ/MMEJ)

Alt-NHEJ/MMEJ is primarily active in the S and G2 phases of the cell cycle and is dependent on signaling by poly(ADP-ribose) polymerase 1 (PARP1) (reviewed in [58]). It relies on 5′–3′ resection of DNA by MRN and CtIP and requires for repair a homologous sequence that is longer than 2 bp. In fact, MMEJ needs longer MHs than NHEJ (2–20 bp versus 0–3 bp) and shorter MHs than single-strand annealing (>15 bp), but the boundaries are overlapping. It is hence complicated to define when MMEJ actually is initiated [59]. Whether MMEJ is a genuine DSB repair pathway is still heavily debated, but it may act as a backup pathway for both NHEJ and HR, when both pathways are overwhelmed with too many DSBs [60]. MMEJ can be roughly distinguished between pre-annealing and post-annealing steps. Prior to annealing of the MH, 5′ to 3′ resection of DNA ends takes place and the resulting 3′ single-strand is able to mediate the MH-based annealing process. Annealed ends are subject to fill-in synthesis by DNA polymerase θ (pol θ), which stabilizes the annealed intermediates and promotes end joining [58]. Post-annealing, the non-homologous tails are removed by the XPF/X-ray repair cross complementing 1 (XPF/XRCC1) nuclease complex and subsequent ligation is carried out by DNA ligase III (LIG3; reviewed in [15]).

### 2.5. Crosslink Repair (Fanconi Anaemia/BRCA Pathway)

The Fanconi anemia (FA) pathway (Figure 1C) is the major pathway for cross-link repair and essentially takes place during the S phase in response to stalled replication forks. The FA pathway combines HR and nucleotide excision repair (NER) and cannot be considered as a discrete DSB repair pathway [61]. One of the major functions of the FA pathway is the stabilization of replication forks and their protection from Mre11-mediated degradation. Mre11, as mentioned above, is part of the MRN complex and with its nuclease activity it is important for end resection in DSB repair [61]. In a very reduced view, the FA pathway can be divided into two major phases: the recognition/signaling phase that is carried out by a first E3 ubiquitin ligase core complex and the proper repair phase that is initiated by the Fanconi anemia complementation group protein D2/BRCA2 (FANCD2/BRCA2) complex [41,62]. These two steps lead to the generation of DSB intermediates that can be repaired by HR [63]. In this respect, FANCD2, once it is mono-ubiquitylated by the core complex, can relocalize to chromatin (Figure 1C) and act together with components of the HR repair pathway, such as NBS1 [64], BRCA1 [65], and Rad51 [66]. All in all, 19 different components implicated in the FA pathway have been identified to date and germline inactivation of any single component leads to the development of Fanconi anemia [61]. A number of heterozygous mutations in FA-related genes are implicated in the susceptibility to several cancers: BRCA1 and BRCA2 mutations are associated with breast and ovarian cancer [46] and similarly Fanconi anemia complementation group protein J (FANCJ) mutations predispose to breast cancer [67]. BRCA2, Fanconi anemia complementation group protein C (FANCC) and a combination of FANCC and Fanconi anemia complementation group protein G (FANCG) mutations have been found in pancreatic cancer patients [68,69].

## 3. Compromised Double Strand Break Repair in Leukemia

Leukemia is a genetically and phenotypically heterogeneous group of diseases, characterized by blocked differentiation and uncontrolled proliferation of hematopoietic precursors cells, of both lymphoid and myeloid lineage [70]. Accordingly, four major types of leukemia can be distinguished: acute myeloid leukemia (AML), acute lymphocytic leukemia (ALL), chronic myeloid leukemia (CML) and chronic lymphocytic leukemia (CLL). These four major groups can be further divided into specific subtypes [70]. Despite the large heterogeneity in leukemia, with respect to the disease-causing mutations, disease progression, treatment and treatment outcome, alterations in DDR, (predominantly in DSB repair), have been shown for each leukemia subtype (Table 1). Some treatment options for leukemia are available to date, but for numerous cases efficient therapy is still lacking and it is hence of outmost importance to understand the underlying cellular defects in these leukemia forms to improve patient prognosis.

### 3.1. Altered Non-Homologous End Joining

**Figure 2:**
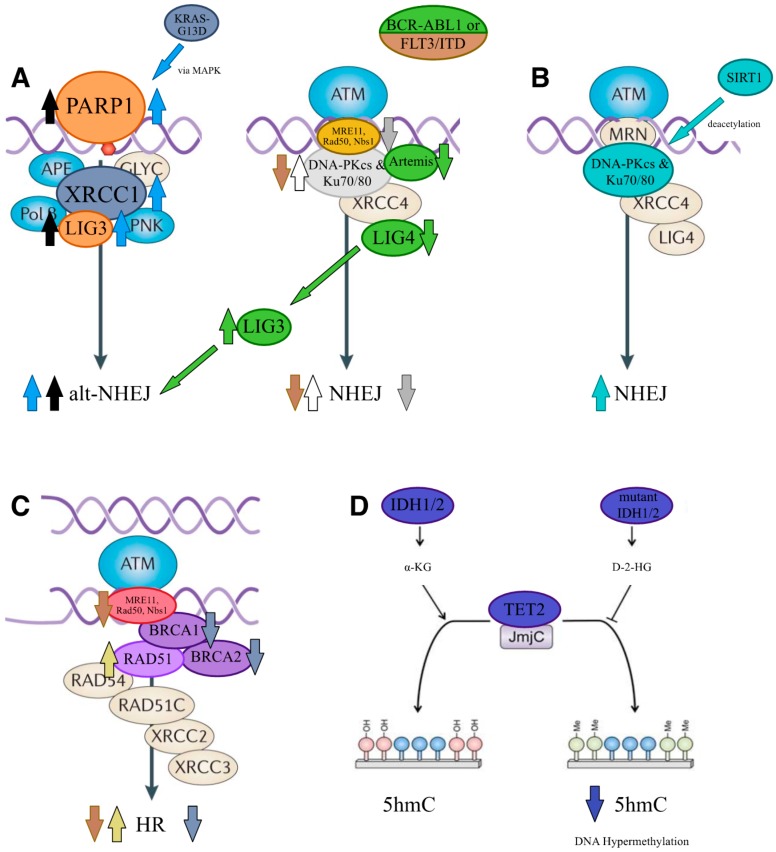
Affected DSB repair components in leukemia. (**A**) Upregulated PARP1 leads to upregulation of DNA ligase III (LIG3) and thus a more active alternative NHEJ (alt-NHEJ) (black arrows). The presence of an oncogenic Kirsten ras oncogene (K-RAS) mutant leads to upregulation of PARP1 and consequently upregulated XRCC1 and LIG3 and more active alt-NHEJ (blue arrows). The presence of the fusion protein between the Rho guanine nucleotide exchange factor (RhoGEF) and GTPase activating protein BCR and the non-receptor tyrosine kinase ABL1 (BCR-ABL1) leads to a decrease of Artemis and LIG4 and consequently LIG3 is upregulated and the repair directed towards alt-NHEJ instead of NHEJ (green arrows). BCR-ABL1 or the colony stimulating factor 1 receptor (CSF1R or FMS)-like tyrosine kinase 3 internal tandem duplication (FLT3/ITD) leads to a decrease in Ku70/80 and DNA-dependent protein kinase catalytic subunit (DNA-PKcs) activity and thus decreased NHEJ (brown arrows). Increased DNA-PKcs activity due to changes in Ku70/80 leads to enhanced NHEJ activity (white arrows). A chromosome deletion affecting MRE11 expression leads to a decrease in both NHEJ and HR (gray arrows; brown arrows in **C**). (**B**) SIRT1 overexpression leads to higher Ku70/80 activity and an increase in NHEJ (blue arrows). (**C**) The presence of BCR-ABL1 provokes increased Rad51 levels, which result in higher HR activity (yellow arrows), whereas downregulation of BRCA1/2 leads to a decrease in HR activity (gray arrows). (**D**) The presence of mutant IDH1/2 or mutated ten-eleven translocation 2 (TET2) leads to reduced 5-hydroxymethylcytosine (5hmC), which indirectly affects DSB repair. Identical colors indicate that the different components are affected together or have an effect on one another. (2**A**–**C**: adapted from [12]; 2**D**: adapted from [93]).

Defects in NHEJ can be observed in all acute and chronic forms of leukemia. Central targets for deregulation in this context are the Ku70/80 complex and DNA-PK. Patients suffering from B-cell lymphocytic leukemia (B-CLL), for example, are typically treated with apoptosis-inducing agents, such as nucleosides, fludarabine and alkylating agents, and chlorambucil [71], but often patients show resistance to treatment. Blaise and colleagues investigated a possible link between treatment-induced apoptosis, DDR and the accumulation of chromosomal aberrations [72]. Their results indicated that an early activated, but unfaithful NHEJ pathway masks the DSB in a way that the apoptotic response is not activated. This leads to the accumulation of chromosomal rearrangements, such as dicentric and ring chromosomes, that are detected in B-CLL cells [72]. In 2002, Gaymes and colleagues recognized increased NHEJ activity in CML and AML patients, as well as in several myeloid cell lines [73]. They showed that this over-activity leads to an unfaithful repair of the DSBs and that the Ku70/80 heterodimer (Figure 2A, white arrows) is somehow implicated in the process [73]. By employing end ligation and plasmid reactivation assays, the authors demonstrated that the end ligation efficiency and the plasmid reactivation, i.e., faithful repair, were decreasing with increasing concentrations of Ku70 or Ku80 antibodies, which is in contrast to what they have observed with DNA-PK antibodies [73]. Resistant B-CLL is associated with high DNA-PK (Figure 2A, white arrows) activity [74,75] and enables B-CLL cells to escape irradiation-induced apoptosis [74]. This increased DNA-PK activity is a consequence of either a variation in the DNA-end binding activity of the Ku heterodimer, the presence of a variant form of it [75], or, as identified in CLL patients, a specific phosphorylated form of Ku70 (i.e., on serine 33) [76].

Decreased levels of DNA-PK were, on the contrary, found in the presence of the fusion protein between the Rho guanine nucleotide exchange factor (RhoGEF) and GTPase activating protein BCR and the non-receptor tyrosine kinase ABL1 (BCR-ABL1) in CML (Figure 2A, brown arrows) [77]. Due to its tyrosine kinase activity, BCR-ABL1 somehow directs DNA-PK to the proteasome without affecting messenger RNA (mRNA) levels of DNA-PK and the expression levels of Ku70/80. Despite the reduced activity of the DNA-PK complex, CML patients accumulate chromosome aberrations as seen in B-CLL patients and the deficiency in DNA repair is counterbalanced by resistance to apoptosis [77]. A decrease in Ku70 and/or DNA-PK, however, is not uniformly seen in patients with BCR-ABL1 fusion [77,78]. Tobin and colleagues therefore hypothesize that instead increased levels of reactive oxygen species (ROS) inhibit Ku70 function, as its end binding activity is blocked by oxidative stress [79]. Consistently, Nowicki and colleagues showed that the presence of BCR-ABL1 in fact leads to elevated ROS levels [80]. Decreased activity of DNA-PK was also found in a promyelocytic (PML) cell line, which in this case arose from a truncated form of Ku80, which reduced the DNA binding activity of the whole DNA-PK/Ku complex [81,82].

Further deregulation of NHEJ in BCR-ABL1-associated CML patients may arise from decreased expression levels of LIG4 and Artemis (Figure 2A, green arrows), without changes in DNA-PKcs [78]. Mutations in LIG4 coinciding with reduced and less efficient NHEJ have also been reported in ALL patients [83]. To compensate LIG4 and Artemis down-regulation, LIG3 together with its interacting DNA helicase WRN is upregulated [78]. Upregulated WRN in CML is constitutively phosphorylated by BCR-ABL1, which inactivates its helicase and exonuclease activity [84]. LIG3, which is typically required for alt-NHEJ (see above) and base excision repair (BER) [85], is also upregulated in the presence of the colony stimulating factor 1 receptor (CSF1R or FMS)-like tyrosine kinase 3 internal tandem duplication (FLT3/ITD) [86]. How reduced LIG4 levels impair NHEJ is not fully understood, but Dn14, the yeast homologue of LIG4, stabilizes Ku70 [87]. If conserved in mammalian cells, one would assume that decreased levels of LIG4 in CML patients would result in destabilized Ku70 and in turn in increased end resection [78]. 

In the mouse proB cell line BaF3, lowered levels of Ku70 and Ku80 (Figure 2A, brown arrows) were detected in the presence of FLT3/ITD [86]. FMS (or CSF1R) is the receptor for colony stimulating factor 1, a cytokine that controls the production, differentiation and function of macrophages (reviewed in [88]). The presence of FLT3/ITD leads to signal transducer and activator of transcription 5 (STAT5)-mediated increased production of ROS, which appears critical for disease onset [86,89]. The increased ROS levels likely provoke an inefficient DSB repair due to the sensitivity of DNA-PKc and the Ku proteins towards oxidative damage [86]. With not much success, FLT3 inhibitors were tested as treatment in phase I/II monotherapy and phase III polytherapy trials [90,91], but these early inhibitors were not specific for FLT3 and inhibited other kinases at high doses [86]. Therefore, a combination of FLT3 inhibition and of targeting the NHEJ pathway was considered as a more promising treatment option for patients with FLT3/ITD-positive AML [86]. Currently, several clinical trials with a newer generation of FLT3/ITD inhibitors are ongoing, either as monotherapy or combinatory therapy. Midostaurin, in combination with induction chemotherapy led to an overall increased survival in patients with FLT3/ITD and other FLT3 mutations. Moreover, also other FLT3 inhibitors of the newer generation, such as quizartinib and gilteritinib, have shown promising results in diverse clinical trials (reviewed in [92]).

Another mode of Ku complex deregulation found in leukemia is by altered acetylation of Ku70. Sirtuin 1 (SIRT1) (Figure 2B, blue arrows) is an NAD+ dependent deacetylase, known to regulate DDR factors such as NBS1 and 53BP1 [94,95]. SIRT1 is also able to deacetylate Ku70, which activates the protein [96], and higher expression of SIRT1 has been observed in CML and AML patients [97]. Zhang and colleagues thus hypothesized that the over-expression of SIRT1 leads to an increase in NHEJ efficiency due to upregulation of Ku70 [97]. Moreover, SIRT1 deacetylates and thus inactivates p53 and forkhead box O1 (FOXO1), two inducers of cell cycle arrest and apoptosis as well as inhibitors of proliferation [97]. Together, these studies indicate that the DNA-PKc and its interacting complex Ku70/80 are the major deregulated factors in NHEJ defects associated with leukemia, in particular CLL and CML. It is important to note that both a decrease and an increase in NHEJ activity are associated with leukemia, underlining the necessity of very tight regulation of this pathway.

### 3.2. Altered Alternative Non-Homologous End Joining

Enhanced alt-NHEJ activity coinciding with increased genomic instability was found in CML patients as consequence of over-expression of PARP1, together with DNA ligase IIIα (Figure 2A, black arrows) [79]. In AML patients, increased alt-NHEJ activity was due to increased expression of PARP2 [98]. PARP1 and PARP2 are involved in BER, which is defined as the removal and replacement of a damaged or mismatched base in the genome as a result of spontaneous deamination, radiation, oxidative stress, alkylating agents or replication errors [99]. The inhibition of PARPs blocks the BER pathway [100] and as a consequence unrepaired SSBs that, when they collide with replication forks, result in DSBs that can no longer be repaired [98]. PARP inhibitors (PARPi), such as olaparib or veliparib, were identified to be effective drugs against leukemia with low expression of the HR factors BRCA1 and BRCA2 [98,101]. In respective AML mouse models for AML1-ETO and PML-RARα fusion proteins, which have decreased expression of HR factors, such as Rad51 and BRCA1, PARPi treatment was also beneficial [101]. The beneficial PARPi effect is suppressed by expression of the homeodomain transcription factor homeobox A9 (HOXA9) [101]. HOXA9 is linked to impaired differentiation of hematopoietic precursor cells and is frequently activated in mixed-lineage leukemia (MLL)-related AML and ALL, T-cell acute lymphoblastic leukemia (T-ALL) and in Nup98-rearranged AML (reviewed in [102]). PARPi, in combination with inhibition of glycogen synthase kinase 3 (GSK3), a serine-tyrosine kinase, is able to counteract the effect of HOXA9 expression [101]. GSK3 mediates the phosphorylation of the cAMP responsive element binding protein (CREB)-binding protein (CBP), an important co-factor for HOXA9 [103]. CBP phosphorylation is required for the transcriptional functions of HOXA9 and thus its inhibition prevents the PARPi suppressive effect of HOXA9. Therefore, HOXA9 might be an attractive therapeutic target for leukemia patients with a constitutive HOXA9 expression, such as MLL-related leukemia, as it is mostly dispensable for normal development of hematopoietic progenitors [101].

Oncogenic K-RAS mutations also direct DSB repair in leukemia towards the error-prone alt-NHEJ pathway. Ras proteins belong to the family of small GTPases and four isoforms of Ras are known in humans: Ki-RAS or K-RAS, N-RAS, Ha-RAS and R-RAS (reviewed in [104]). Ras proteins regulate gene expression due to interaction with the Raf protein kinase, which induces the mitogen-activated protein/extracellular signal regulated kinase (MAP/ERK) kinase (MEK)/MAP kinase signaling pathway (reviewed in [104]). Mutations in Ras are found in around 30% of all human cancers [104]. Cells often become strictly dependent on the mutant Ras protein and Ras-mutated cancers are associated with poor treatment outcome [105]. Expression of an oncogenic K-RAS (K-RASG13D) (Figure 2A, blue arrows), a constitutively guanosine-diphosphate (GDP)-bound form of K-RAS, in T-ALL and AML cell lines leads to a delay in DSB resolution and a more error-prone repair [106]. The cells are preferentially directed towards the alt-NHEJ pathway via the MAPK pathway, coinciding with an increase in DNA ligase IIIα, PARP1 and XRCC1, all of which are components of the alt-NHEJ pathway [106]. Oncogenic K-RAS thus directs the DSB repair toward the error-prone alt-NHEJ pathway, so that the pharmacological inhibition of components of this pathway would constitute a possible therapeutic target [106].

### 3.3. Altered Homologous Recombination

The key facilitator of homologous recombination is Rad51 (see above) and Christodoulopoulos and colleagues found in 1999 that Rad51 (Figure 2C, yellow arrows) is activated by the alkylating agent chlorambucil (CLB) used in chemotherapy in B-CLL patients [107]. The HR repair pathway was not well characterized at that time, but the authors suspected that Rad51 played a role in the HR pathway and proposed it as a possible therapeutic target in B-CLL patients [107]. Enhanced DSB repair due to over-expression of Rad51 mRNA and protein has been observed in CML patients [108]. This increased expression is caused by the BCR-ABL1-mediated activation of the transcription factor STAT5. Rad51 is a known substrate for caspase-3 [109], and BCR-ABL1 expression leads to further increased Rad51 expression levels due to the inhibition of caspase-3 activation [108]. Rad51 has numerous, non-redundant paralogs that act together during HR repair. Among these paralogs hRad51B, hRad51D, and hXRCC2 were also found upregulated in CML. A third aspect is that both the non-receptor tyrosine kinase ABL proto-oncogene 1 (c-abl) and BCR-ABL1 directly interact with Rad51 and are responsible for its phosphorylation. As BCR-ABL1 is constitutively active, Rad51 is constitutively phosphorylated [108]. Richardson and colleagues showed that a transient over-expression of Rad51 leads to genome instability due to a shift from HR to more error-prone repair, namely the aberrant combination of SSA and NHEJ [110]. Genetically engineered 32Dcl3 cells expressing BCR-ABL1 show an increased expression of Rad51 and are resistant to cisplatin and mitomycin C treatment [108]. Cisplatin induces inter- and intrastrand crosslinks that are refractory to excision and thus often block replication forks and induce DSBs [111]. Increased Rad51 expression in cells derived from CML patients were subsequently demonstrated by Slupianek and colleagues [108]. An increased risk for de novo and treatment-related AML (t-AML) has been associated with the presence of polymorphisms in several HR genes: *RAD51*-135C and its paralog *XRCC3*-241Met [112].

Down-regulation of BRCA1/2 has been observed in AML patients and resulted in defective HR repair (Figure 2C, gray arrows) [98,113]. Scardocci and colleagues found that the decreased expression of BRCA1 was due to the hypermethylation of its promoter by the DNA methylase DNMT3A [113]. Down-regulation of BRCA1 is also a characteristic of BCR-ABL1-related CML [114]. How BRCA1 downregulation is mediated has remained unclear in this case, but the tyrosine kinase activity of BCR-ABL1 is therefore of importance, similar to what has been observed for DNA-PK decrease in CML [114]. Last but not least, Mre11A and ATM (Figure 2C, brown arrows) can be affected by a deletion of chromosome 11 in t-AML patients (see below), which leads to alterations in both NHEJ and HR as Mre11A is an early factor in these two pathways [115].

### 3.4. Altered Single-Strand Annealing

Alterations in SSA have been described in CML patients. The presence of BCR-ABL1, but also other fusion tyrosine kinases, such as erythroblast transformation specific (ETS) variant 6 (ETV6 also known as TEL)–Janus kinase 2 (TEL-JAK2) and TEL-ABL1, stimulate the SSA activity [116]. In the same context, Mattarucchi and colleagues showed in 2008 that the mechanism involved in the t(9:22) translocation leading to BCR-ABL1 is often due to SSA and NHEJ [117]. The presence of BCR-ABL1 in 32Dcl3 leads to increased ROS levels and consequently increased DSBs and they were shown to be repaired by SSA [116].

### 3.5. Alterations in Other Repair Pathways

While in most cases of defective DDR in leukemia response to DSBs is impaired, in a few cases defects in BER were also described. As mentioned earlier an upregulation of LIG3 is typically observed when LIG4 is affected [78]. This leads to increased alt-NHEJ, but whether this has also an effect on the BER pathway has not been studied yet. Alterations in the FA pathway have also been observed, which as a first consequence results in the development of Fanconi anemia, but about 9% of patients subsequently develop mostly myeloid leukemia with a high incidence of chromosomal breakage [118]. Heterozygous deletions and distinct point mutations in the Fanconi anaemia complementation group protein A (*FANCA*) gene were found in a small percentage of AML patient samples [119,120]. In a T-ALL patient, a *FANCC* point mutation was identified [121]. However, since patient numbers were rather small, a larger cohort study is required to more significantly determine whether *FANCA* and *FANCC* mutations are commonly implicated in AML and/or T-ALL [119].

### 3.6. Indirect Effects on DDR

Several leukemia-related mutations affect chromatin structure or gene regulation and thus indirectly DDR. In this context, changes in isocitrate dehydrogenase 1 and 2 (*IDH1/2*) and in the dioxygenase ten-eleven translocation 2 (*TET2*) (Figure 2D) are driver mutations in myeloid malignancies, but the underlying mechanisms remain rather obscure [122,123]. Heterozygous, mutually exclusive missense mutations of IDH1/2 have been identified in AML [121], for example the R140G IDH2 mutant in myeloid cell lines [122]. *TET2* mutations occur in around 50% of chronic myelomonocytic leukemia (CMML), a subtype of myelodysplastic syndrome/myeloid proliferative neoplasm (MDS/MPN) [124], as well as in around 30% of myeloproliferative neoplasms and AML [125]. Mutations in *IDH1/2* and *TET2* lead to a decrease in 5-hydroxymethylcytosine (5hmC) and consequently to DNA hypermethylation. DNA hypermethylation is in part due to the inhibition of TET2 and the dioxygenases of the AlkB homolog (ALKBH) family, which are Fe (II) and α-ketoglutarate (α-KG)-dependent dioxygenases [126] that repair alkylated DNA [124,127]. When *IDH1/2* are mutated, AKLBH enzymes lose their normal catalytic activity and gain activity, which leads to the reduction of α-KG into antagonistic D-2-hydroxyglutarate (D-2-HG), which in turn inhibits the AKLBH dioxygenases [126,128]. The presence of D-2-HG induces increased ROS production and further inhibits the ALKBH enzymes and the repair of alkylated DNA [126]. The cells are thus hyper-sensitive to alkylating agents and are characterized by the presence of increased DSBs, which result from the unrepaired DNA alkylations [126].

Small molecule inhibitors of IDH1/2 are consequently of high interest as possible therapeutic drugs. It is not clear yet whether these inhibitors will have an overall positive effect on their own or whether they sensitize the cells towards an additional treatment. Besides the direct inhibition of IDH1/2, demethylation of DNA and histones is another treatment option. Two DNA demethylating agents, 5-azacitidine and decitabine, have already been used for different AML subtypes and could be of interest in IDH1/2-mutant leukemia (reviewed in [123]). Reactivation of genes at silenced chromatin can further be achieved by using histone deacetylation inhibitors (HDACi) and in fact several HDAC inhibitors have been approved as blood cancer therapy in the last 15 years. Vorinostat and romidempsine are effective against cutaneous T-cell lymphoma [129], belinostat has been approved for the treatment of peripheral T-cell lymphoma [130], and panobinostat in combination with bortezomib and dexamethasone for therapy of multiple myeloma [131].

Well-known cancer key players, such as the proto-oncogene MYC and p53, are also involved in leukemia and defective DSB repair, although comparatively little. MYC was found over-expressed in t-AML and in de novo AML patients due to a trisomy of chromosome 8, the chromosome on which the *MYC* gene is located [115]. MYC target genes, such as cyclins A2, D2 and E1, cyclin-dependent kinases, ribosomal proteins and nucleophosmin [132] are upregulated in these patients just as ROS [115]. High MYC expression leads to elevated, persistent DSBs in primary mouse hematopoietic myeloid progenitors [115]. In B-CLL, about 10% to 15% of cases are related to either structural alterations, gene deletions or point mutations in p53 [71,72,133] and this is similar in ALL patients [134,135]. B-CLL patients with mutant p53 have a low survival rate and show high therapy resistance [72]. In t-AML, p53 aberrations are one of the most common mutations [136]. The patients show high genomic complexity and complex karyotypes, which altogether lead to an inferior survival rate. TP53 alterations lead to slower DSB resolution, but the exact role of p53 in this context has remained elusive [115,136]. P53 function may further be indirectly impaired due to dysfunctional ATM, one of the key players early in the DSB signaling (see above). In 10% to 20% of CLL, patients show a deletion of the long arm of chromosome 11, which appears to be related to a poor disease outcome. ATM is one of the genes affected by this deletion. The presence of one functional allele of ATM is sufficient for the activation of the p53/p21 pathway, but a mutant allele is not, which is associated with a shorter survival time of the patients. Chemotherapeutic treatment exhibits a certain selective pressure on the remaining ATM allele, so that the use of a therapy by-passing the ATM/p53 pathway might be more beneficial for patients [137].

## 4. Conclusions

Genomic integrity is of outmost importance and key to human health and it is maintained by DNA damage response. Particularly challenging for genome integrity are DNA double-strand breaks and defects in their repair are frequently observed in leukemia. DSB repair signaling and pathways are complex, but frequent targets for interfering with faithful DSB repair appear to evolve: the DNA-PK and Ku70/80 complex in the NHEJ pathway and Rad51 and BRCA1/2 in the HR pathway. The identification of this decisive factors will broaden the therapeutic spectrum in leukemia for general, combinatory therapy. Moreover, in combination with approaches towards identification of the specific disease signature for each leukemia subtype, knowledge of which DSB factor would be the best therapeutic target is of outmost importance to shift away from general broad-spectrum treatment, towards personalized therapy. As DSB repair pathways are somewhat interconnected, this interconnection can be used to develop synthetic lethality-based targeted therapies [61], which would be of tremendous benefit for patients with poor treatment response and poor prognosis.

## Figures and Tables

**Table 1:** DNA repair pathways compromised in leukemia.

Repair Pathway	Affected Component	Leukemia Subtype
Non-homologous end joining	DNA-PK, Ku70/80	B-CLL, CML, AML, CLL, PML
	DNA ligase IV, Artemis	CML
	Mre11A	t-AML
	SIRT1	CML, AML
Homologous recombination	BRCA1/2	CML, AML
	Rad51	B-CLL, CML, de novo and t-AML
	Mre11A	t-AML
Alternative non-homologous end joining	K-RAS	T-ALL, AML
Fanconi anemia	FANCA	AML
	FANCC	T-ALL
Base excision repair	PARP1/2	CML, AML
	DNA ligase III	CML
Non-specific	IDH1/2	CMML, AML
	ATM	CLL
	MYC	t-AML
	TP53	B-CLL, ALL, t-AML

DNA-PK: DNA-dependent protein kinase; SIRT1: sirtuin 1; BRCA1/2: breast cancer 1/2; K-RAS: Kirsten ras oncogene; FANCA: Fanconi anemia complementation group protein A; FANCC: Fanconi anemia complementation group protein CC; PARP1/2: poly(ADP-ribose) polymerase 1/2; IDH1/2: isocitrate dehydrogenase 1/2; ATM: Ataxia telangiectasia mutated; MYC: myc proto-oncogene; B-CLL: B-cell lymphocytic leukemia; CML: chronic myeloid leukemia; AML: acute myeloid leukemia; PML: promyelocytic leukemia; t-AML: therapy-related acute myeloid leukemia; T-ALL: T-cell acute lymphocytic leukemia; CMML: chronic myelomonocytic leukemia.
